# Mycobacteria in Water Used for Personal Hygiene in Heavy Industry and Collieries: A Potential Risk for Employees

**DOI:** 10.3390/ijerph120302870

**Published:** 2015-03-04

**Authors:** Vit Ulmann, Anna Kracalikova, Radka Dziedzinska

**Affiliations:** 1 Institute of Public Health, Partyzanske namesti 7, 702 00 Ostrava, Czech Republic; E-Mail: Anna.Kracalikova@zuova.cz; 2 Veterinary Research Institute, Hudcova 70, 621 00 Brno, Czech Republic; E-Mail: dziedzinska@vri.cz

**Keywords:** environmental mycobacteria, surface water, mining water, shower, industry, colliery

## Abstract

Environmental mycobacteria (EM) constitute a health risk, particularly for immunocompromised people. Workers in heavy industry and in collieries represent an at-risk group of people as their immunity is often weakened by long-term employment in dusty environments, frequent smoking and an increased occurrence of pulmonary diseases. This study was concerned with the presence of EM in non-drinking water used for the hygiene of employees in six large industrial companies and collieries. Over a period of ten years, 1096 samples of surface water treated for hygiene purposes (treated surface water) and treated surface water diluted with mining water were examined. EM were detected in 63.4 and 41.5% samples of treated surface water and treated surface water diluted with mining water, respectively. *Mycobacterium gordonae*, *M. avium-intracellulare* and *M. kansasii* were the most frequently detected species. Adoption of suitable precautions should be enforced to reduce the incidence of mycobacteria in shower water and to decrease the infectious pressure on employees belonging to an at-risk group of people.

## 1. Introduction

For financial reasons, large companies often use surface-treated water from neighbouring rivers or retention basins for the hygiene of their employees. The usage of surface water for this purpose is allowed by the law, however, such usage must fulfil the legislative requirements of each individual country. According to the legislation of the Czech Republic [1], surface water treated by companies for their own purposes represents a unique category of water, in which additional parameters, such as mycobacteria, must be controlled. In general, surface water treatment within companies includes sedimentation, sand filtering and chlorination. Mining water is highly mineralized deep-pumped mining water. It is collected during the mining process and must be drained off for work and safety reasons. Mining water can either be released into local rivers or it can be recycled and used for the needs of the company, e.g., as water for personal hygiene. Subsequently, mining water is mixed with surface water and they are treated together.

The environmental mycobacteria group (EM; non-tuberculous mycobacteria, atypical mycobacteria) consists of a large group of microorganisms ranging from common saprophytes to opportunistic pathogens present in ubiquitous matrices, such as soil, dust or water. The group includes tens of species, but *Mycobacterium avium*, *M. fortuitum*, *M. chelonae*, *M. kansasii*, *M. gordonae* and *M. xenopi* are the most commonly described, especially in water [2,3]. EM exhibit considerable tolerance to unfavourable conditions and are able to survive aggressive treatments like chlorination or ozonation of water [4,5]. EM are opportunistic pathogens and are transmitted to humans via inhalation, ingestion, or inoculation [6]. Immunocompromised individuals are particularly at risk for infection [2]. Although such individuals do not typically work in mines or in heavy industry, EM can also represent a high risk for ordinary employees. In particular, miners in collieries are exposed to highly dusty environments, often work in mines over their entire working life and are frequently heavy smokers. Further, some of these workers develop chronic obstructive pulmonary disease. The high occurrence of EM in the environment together with the impaired immunity of the lungs of exposed individuals can lead to colonization and infection.

The aim of this paper was to examine two non-drinking types of water (treated surface water and treated surface water diluted with mining water) used for personal hygiene in large industrial companies and collieries for the presence of EM. In the case of industrial companies and collieries whose employees represent an at-risk group of people (impaired lung immunity), our observation has additional implications. Previously, many studies have revealed the presence of EM in water distribution systems in households and hospitals [7,8,9,10]. Water therapy pools and swimming pools were also described as sources of EM [11,12]. Ground, surface or rain water intended for human use was less frequently studied [4,13,14]. To our knowledge, no published report has examined surface water treated for the hygiene of people and not intended for public drinking water distribution systems for the presence of EM.

## 2. Experimental Section

### 2.1. Origin of the Samples

During ten consecutive years, 1096 samples of water intended for hygiene were collected from six industrial companies (heavy industry companies, steel mills and coal mines) located in the northern part of the Moravia region in the Czech Republic. The bulk of the samples (954) were treated surface water diluted with mining water and 142 were treated surface water. Reservoirs and rivers in the proximity of industrial companies were used as a source of surface water. The treatment of surface water included sedimentation, sand filtration and chlorination. The treated water was then stored either in tanks where it could be directly heated or it was heated by hot water heaters within the companies.

One litter of each sample was collected from terminal parts of the distribution system (tap or shower) into sterile bottles. Water was taken after flushing the tap or showerhead for one minute. The samples were processed within 24 h (transportation at 4 °C).

### 2.2. Processing of Water Samples

The cetylpyrimidium chloride (CPC) method was carried out as described by Neumann *et al.* [15] by adding CPC (cetylpyrimidium chloride monohydrate, Merck Schuchardt OHG, Hohenbrunn, Germany) to the samples to give a final concentration of 0.005% (w/w) and shaking of the mixture for 30 s. After 30 min of exposure, the samples were filtered using the Millipore filtration system (vacuum pump WP6122050, membrane filters no. HAWG04796, 47 mm, pore size, 0.45 µm; Merck Millipore, Billerica, MA, USA) and rinsed with 100 mL of sterile water to remove residual CPC. The filters were transferred to centrifuge containers with 2 mL of distilled water and approx. 2 g of sterile glass beads (2 mm; Kavalierglass, Sazava, Czech Republic) and shaken vigorously by vortexing for 5 min.

The samples were inoculated onto Lowenstein-Jensen media (home-made). Incubation was carried out at three different cultivation temperatures (30, 37 and 42 °C) in order to facilitate the detection of mycobacterial species with different growth optima. Six cultivation tubes were prepared for each sample (duplicates for each temperature). Results were assessed for up to nine weeks after the inoculation. From 2001 to 2005, all samples were evaluated using microscopy, cultivation and phenotypic methods. From 2006 onwards, the above-mentioned methods were supplemented with molecular biology methods, such as the GenoType^®^ Mycobacterium CM/AS (Hain Lifescience GmbH, Nehren, Germany), and AccuProbe RNA hybridization assays (Gen-Probe Inc., San Diego, CA, USA).

### 2.3. Statistical Assessment

Statistical analysis was performed using the statistical software GraphPad Prism 5.04 (GraphPad, Inc., San Diego, CA, USA). The frequency of occurrence of positive samples was compared using Fisher’s exact test. The distribution of mycobacterial species in different types of water was analyzed using the Chi-squared test for independence and standardized Pearson’s residuals. *P*-values lower than 0.05 were considered to be significant in all cases.

## 3. Results

**Figure 1:**
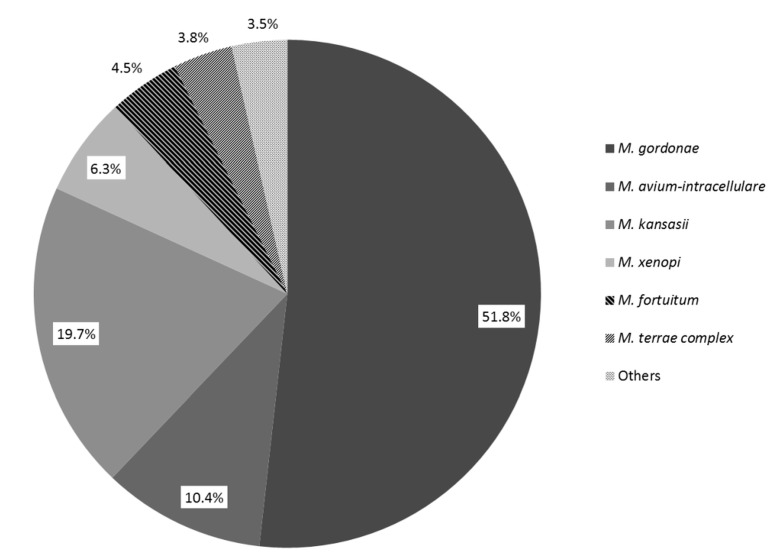
The proportions of environmental mycobacteria detected in treated surface water diluted with mining water intended for the hygiene of employees in large industrial companies and collieries.

**Figure 2:**
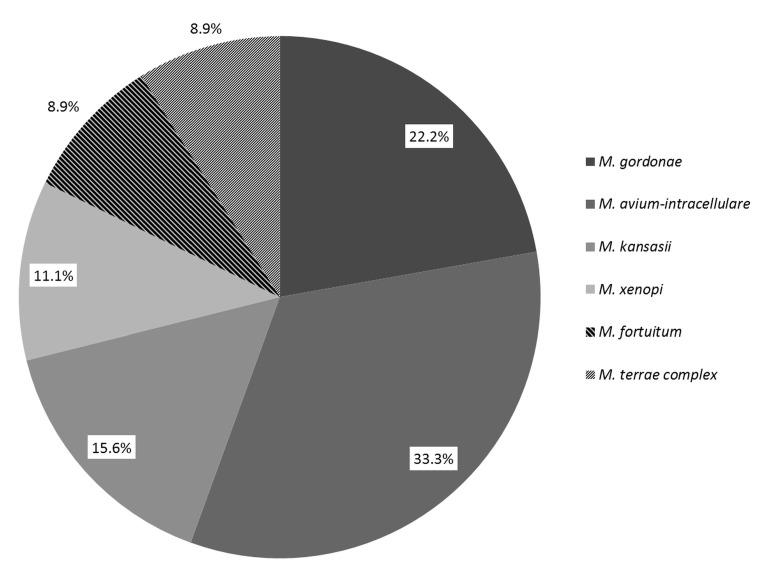
The proportions of environmental mycobacteria detected in treated surface water for the hygiene of employees in large industrial companies and collieries.

From the set of collected samples, 41.5% (396 from 954) and 63.4% (90 from 142) of treated surface water samples diluted with mining water and treated surface water samples were found to be EM-positive, respectively. *M. gordonae* was the predominant species recovered from mining water-diluted samples (51.8%; Figure 1). In treated surface water, *M. avium-intracellulare* was found to be predominant (33.3%; Figure 2).

Besides the two above-mentioned species, *M. kansasii* numbered among the three most commonly recovered species in all types of water (Figure 1 and Figure 2). Apart from the EM species commonly found in water (Figure 1 and Figure 2), the presence of others, such as *M.* *mucogenicum*, *M. simiae*, *M. lentiflavum*, *M. interjectum*, *M. scrofulaceum*, *M. chelonae* and *M. flavescens* was also demonstrated. All of these were detected only in the case of surface water diluted with mining water and numbered about one to three positive samples per species over the whole examined period (0.1%–0.2%).

## 4. Discussion

Although raw natural water is a primary source of mycobacteria, the CFU numbers are generally very low upon entrance to water distribution systems. However, after storage and heating in water systems, mycobacteria reproduce, leading to a significant increase in their numbers, thus enabling their detection. Unlike drinking water, water for industry use must legislatively fulfill health criteria for non-potable water only [1]. This is reflected in the process of its treatment which is considerably less demanding compared to drinking water (and indicated by frequent findings of diatom frustules, algae, and nonspecific microflora when using microscopy). In contrast, other parameters, such as the presence of mycobateria have to be determined in treated surface water but not in drinking water [1].

In treated surface water the positivity was about 20% higher compared to treated surface water diluted with mining water (63.4% *vs.* 41.5%, resp.). This finding is statistically significant (*p* < 0.01; Fisher’s exact test). The occurrence of a positive sample was 2.4-fold higher in samples of non-diluted surface water than in surface water diluted with mining water (OR = 2.439; 95% confidence interval is from 1.693 to 3.513). Mining water similarly as groundwater should be almost sterile compared to surface water [16]. Schwartz *et al.* [17] showed that biofilms from drinking water conditioned from groundwater were less heavily contaminated by mycobacteria than biofilms from bank-filtered drinking water. The reason for our observation may thus lie in simple dilution leading to a reduction in the number of viable mycobacteria in treated water diluted with mining water. The presence of uncultivable mycobacteria in ground waters can also contribute to the lower number of mycobacteria detected in this environment [2].

In general, the isolated mycobacteria represented species with a wide spectrum of growth temperatures. Thus, more adaptable mycobacterial species, for which the primary temperature of incoming water and relatively low heating temperatures are suitable, were identified. The majority of samples collected from surface water diluted with mining water contained *M. gordonae* as the dominant mycobacterial species (Figure 1), while other species were detected less frequently. In surface, non-diluted water, *M. avium-intracellulare* was predominant, followed by *M. gordonae* (Figure 2). In both types of water, the different distribution of the predominant species (*M. gordonae* or *MAI*) was statistically significant (*p* < 0.01).

Compared to other mycobacteria, *M. gordonae* is very well adapted to survive in artificial environments, such as water distribution systems. Although present in all parts of water systems, the highest number of *M. gordonae* were found at the most distal sites of lines, where the levels of chlorine were minimal [18]. Additionally, the proliferation of *M. gordonae* in the unheated lines (below 19 °C) was also demonstrated. From the results of the mentioned study [18] it could be supposed that while thermotolerant *M. avium* complex (MAC) species rather inhabit heated parts (heater,storetanks), *M. gordonae* would predominate in the free niche at the distal parts of waterworks (showerheads). Within our study, the presence of *M. gordonae* was demonstrated in all positive samples, alone or in mixed cultures with other species. It could be supposed that when mining water (almost sterile) was used the number of mycobacteria (mainly MAI) coming from surface water into the distribution system was diluted. This dilution, which would lead to decreased numbers of competitors could probably account for the higher prevalence (dominance) of *M. gordonae*, which normally rather occupy distal parts of water system, e.g., showerheads. When surface water was not diluted, the CFU counts of other mycobacteria entering into the system were most likely sufficient to successfully compete with *M. gordonae* (esp. *M. avium* which is highly resistant to chlorine).

While *M. gordonae* is regarded as the least pathogenic EM, *M. avium-intracellulare* is responsible for a significant number of mycobacterial diseases in immunocompromised people. We are aware that *M. avium-intracellulare* represents two separate mycobacterial species which belong to the MAC. Nevertheless, for monitoring the health risk of non-drinking water intended for hygiene and in agreement with the valid legislation, differentiation into individual species is not relevant. Both MAC species are primarily pulmonary pathogens and are responsible for the majority of mycobacterial lung diseases in immunocompromised persons [2]. Due to the increase in risk factors in people working in heavy industry and in collieries (long-term exposure to dusty environments, smoking, *etc.*), the finding of MAC in water intended for showering is significant and can represent a risk for these people.

Among the three most frequently detected mycobacteria, the presence of *M. kansasii* was also demonstrated in both types of water (Figure 1 and Figure 2). *M. kansasii* is primarily a pulmonary pathogen and is characteristically found in urban regions [2,19,20]. In sporadic cases, *M. mucogenicum, M. simiae, M. lentiflavum, M. interjectum, M. scrofulaceum, M. chelonae and M. flavescens* were detected in treated surface water diluted with mining water. Their detection in markedly low amounts most likely correlates with the seasonal fluctuations in their persistence in the original surface water. It should also be mentioned that methods enabling the discrimination of these species could not be employed between 2001 and 2005.

## 5. Conclusions

The data collected over a ten-year period show considerable EM contamination of industrial water directly used for the hygiene of employees. Considering the risks associated with these groups of employees who are exposed to such water (heavy industry workers, miners), this finding is significant. Therefore, suitable precautions should be adopted to decrease the incidence of EM in industrial companies. This includes common and known procedures, such as changing the disinfection agent used for water treatment or its concentration, or simply a change of the showerheads. Due to the well-known resistance of mycobacteria to disinfection agents and their ability to quickly colonize even new pipes, the consistent and proper application of the above-mentioned precautions would play the most important role in minimizing EM levels. Regular application of such precautions would be expected to decrease the infectious pressure on the employees working in such companies.
